# HIN4/397: Designing the Web- and Fax-based Consultancy and Information Service "EcgConsult" for Clinicians Dealing with ECG Diagnostics and Rhythmologic Problems

**DOI:** 10.2196/jmir.1.suppl1.e35

**Published:** 1999-09-19

**Authors:** C Elsner, G Hindricks, H Kottkamp

**Affiliations:** ^1^Heart Center LeipzigLeipzigGermany

**Keywords:** Medicine, Information system, Fax, Internet, Teleconsulting, Cardiac Arhythmias, ECG, EcgConsult

## Abstract

**Introduction:**

Modern strategies for the diagnosis and treatment of cardiac arhythmias include besides non-invasive methods an increasing variety of invasive and curative therapeutic strategies. In order to define proper rhythmologic diagnosis and to develop an optimal treatment strategy for individual patients, a high degree of rhythmologic expertise is necessary. Adequate platforms for information exchange, where physicians can meet rhythmologic experts to allow proper handling of individual rhythmologic problems are desirable. Thus, the Heart Center Leipzig develops the Web- and Fax-based consultancy and information service "EcgConsult".

**Methods:**

The service utilizes the network capabilities of the Internet and of the common telecommunication network. This way information input can be achieved either by using a browser/email-client or by using a fax machine. All input from both networks is collected on a centralised mail-server (SSL-POP3 protocol). User interaction on the Web site is achieved with dynamic HTML and a moderated List-Server. The List-Server and an additional discussion area on the Web site are moderated by the Consultants at the Heart Center Leipzig, adequate processing of the site-content and technical management of the site have been outsourced to the university Internet workgroup Elchsoft/Medkonsult.

**Results:**

A prototype of the site and the communication architecture for fax/email have been established and successfully beta-tested. Any consulting physician can easily send ECGs and additional material to the server with any fax-machine, where they are automatically TIFF-file converted. A password-protected site is already in use with selected clinicians (http://www.ecgconsult.de). For mobile site management, a web-based Control-Center has been designed. Outlines of contract models for members, guidelines for security purposes of the web-based communication, a business/cost plan and models for the logistic request management as well as for member/patient identification have been developed.

**Discussion:**

The platform "EcgConsult" provides a network with fast and easy access to a specific target group and thus allows easy information exchange between the Heart Center Leipzig and rhythmologic experts and non-experts. This exchange may generate synergy effects by offering both groups a variety of data and information. Due to the use of web- and fax-based technology, the access to the service is easy and does not require PC-equipment or a scanner. The requests can be answered by the Heart Center consultants from any remote location world-wide. For the future open-ended architecture allows the participation of further consultants from clinics all over the world. The knowledge gathered from this project may help setting up guidelines for the development of other web-based medical competence centers / information services.

